# Fundal Height Growth Curve for Underweight and Overweight and Obese Pregnant Women in Thai Population

**DOI:** 10.1155/2013/657692

**Published:** 2013-12-19

**Authors:** Jirawan Deeluea, Supatra Sirichotiyakul, Sawaek Weerakiet, Rajin Arora, Jayanton Patumanond

**Affiliations:** ^1^Clinical Epidemiology Program, Faculty of Medicine, Chiang Mai University, Chiang Mai 50200, Thailand; ^2^Department of Obstetrics and Gynecology Nursing, Faculty of Nursing, Chiang Mai University, Chiang Mai 50200, Thailand; ^3^Department of Obstetrics and Gynecology, Faculty of Medicine, Chiang Mai University, Chiang Mai 50200, Thailand; ^4^Department of Obstetrics and Gynecology, Faculty of Medicine, Ramathibodi Hospital, Mahidol University, Bangkok 10400, Thailand; ^5^Department of Obstetrics and Gynecology, Lampang Regional Hospital, Lampang 52000, Thailand; ^6^Clinical Epidemiology Unit & Clinical Research Center, Faculty of Medicine, Thammasat University, Pathum Thani 12120, Thailand

## Abstract

*Objectives*. To develop fundal height growth curves for underweight and overweight and obese pregnant women based on gestational age from last menstrual period and/or ultrasound. *Methods*. A retrospective study was conducted at four hospitals in the northern part of Thailand between January 2009 and March 2011. Fundal height, gestational age, height, and prepregnancy weight were extracted from antenatal care and delivery records. Fundal height growth curves were presented as smoothed function of the 10th, 50th, and 90th percentiles between 20 and 40 weeks of gestation, derived from multilevel models. *Results*. Fundal height growth curve of the underweight was derived from 1,486 measurements (208 women) and the overweight and obese curve was derived from 1,281 measurements (169 women). The 50th percentile line of the underweight was 0.1–0.4 cm below the normal weight at weeks 23–31 and 0.5–0.8 cm at weeks 32–40. The overweight and obese line was 0.1–0.4 cm above the normal weight at weeks 22–29 and 0.6–0.8 cm at weeks 30–40. *Conclusions*. Fundal height growth curves of the underweight and overweight and obese pregnant women were different from the normal weight. In monitoring or screening for abnormal intrauterine growth in these women, fundal height growth curves specifically developed for such women should be applied.

## 1. Introduction

A demographically specific fundal height (FH) growth curve derived from local pregnant women with specific ethnicity, socioeconomics, or nutritional status [1] is likely to be suitable for monitoring and screening abnormal intrauterine growth in developing countries, especially in areas where ultrasound is not available. It is simple, convenient, safe, inexpensive [2–4], and may reduce transferring rate and may avoid unnecessary ultrasound [5].

However, previous findings showed that in women of the same geographical areas, there were still other independent determinants of FH. These determinants included maternal height, maternal weight, body mass index (BMI), parity, fetal sex, and gestational age (GA) [6–8]. The determinant that most influenced the difference in the pattern of FH growth curve was body shapes of pregnant women (obese-slim or large-small BMI). Given the same GA, FH of obese women was 2 cm higher than that of slim women [7, 8].

Application of FH growth curve derived from “general” population to monitor or screen abnormal intrauterine growth in obese or slim women may result in over- or underinvestigation and/or intervention. Applying separate FH growth curves specific for women body shapes may be more beneficial [7].

In Thailand, separate FH growth curves according to prepregnant BMI, <20, 20–24, and >24 kg/m^2^, and GA assessed from ultrasound [7], were proposed. However, BMI categorization and GA assessment were not based on routine antenatal care practice in most hospitals, in which categorizing BMI followed world health organization (WHO), <18.5, 18.5–24.9, 25.0–29.9, and ≥30.0 kg/m^2^ [9], and GA was routinely assessed by last menstrual period (LMP) and/or ultrasound, based on individual judgment [1]. The proposed FH growth curves were therefore unlikely to be used in routine practice.

The present study aimed to develop fundal height growth curves for underweight (BMI < 18.5 kg/m^2^) and overweight and obese (BMI ≥ 25.0 kg/m^2^) pregnant women in the northern part of Thailand, based on GA from LMP and/or ultrasound following routine practice.

## 2. Subjects and Methods

### 2.1. Pregnant Women

Antenatal care (ANC) and delivery records of women between January 2009 and March 2011 were retrospectively collected from two secondary care and two tertiary care hospitals in the northern part of Thailand.

The study included women whose GA was less than 20 weeks when attending the first ANC visit. The following pregnant women were excluded: non-Thai, minority groups, unreliable GA, those with comorbidity, current smokers, those who used alcohol or addictive substance during pregnancy, those who developed medical complication during pregnancy: diabetes, hypertension, and anemia, those who had twins, uterine tumor, polyhydramnios, oligohydramnios, intrauterine growth restriction, abnormal fetal presentation, preterm or postterm, low birth weight (<2,500 g) or relatively large baby (≥4,000 g), or congenital anomaly.

### 2.2. Prepregnancy Body Mass Index

Prepregnancy BMI was calculated from prepregnancy body weight (in kg) divided by square of height (in meter).

Pregnant women were categorized into 3 groups based on WHO criteria [9]. Obese (BMI ≥ 30.0 kg/m^2^) and overweight (BMI 25.0–29.9 kg/m^2^) women were combined, as follows.

1. Underweight: BMI < 18.5 kg/m^2^.
2. Normal weight: BMI 18.5–24.9 kg/m^2^.
3. Overweight and obese: BMI ≥ 25.0 kg/m^2^.

### 2.3. Ascertainment of Gestational Age

Gestational age was assessed from 2 sources: (1) based on first day of LMP in women with regular menstruation history, who could recall the exact date and those whose FH was correlated with GA, or GA by LMP was no more than 1 week different from ultrasound, (2) from ultrasound performed in the first half of pregnancy, in women who did not fulfill criteria (1).

### 2.4. Fundal Height Measurement

The measurement of FH followed routine practice of the four settings, which was adopted from ANC practice recommended by The Division of Maternal and Child Health and The Ministry of Health. All measurements were conducted by nurses or physicians in ANC clinics who had at least 2 years of experiences. This was based on the finding that such experiences reduced measurement errors and bias [10].

### 2.5. Data Collection and Data Sources

Key information included GA, FH, height, and prepregnancy weight. All information was extracted from ANC records, delivery records, and other related medical records.

### 2.6. Statistical Analysis

Data analysis was done considering the differences of GA calculated by different methods and the differences in FH measurements by different settings and by standardization methods.

The general characteristics of pregnant women were presented by frequency, percentages, mean, and standard deviation. Nonparametric tests for trend were applied to test the differences among the 3 BMI groups.

The mean FH (cm) for each gestational week between underweight and normal weight and between overweight and obese and normal weight pregnant women was compared by *t*-tests.

Polynomial equations of the 10th, 50th, and 90th percentiles of FH on GA among the underweight, normal weight, and overweight and obese pregnant women were conducted by multilevel models for continuous data. Smoothed curves were drawn from final quadratic regression models.

### 2.7. Ethical Approval

The study protocol was approved by the Research Ethics Committee, Faculty of Medicine, Chiang Mai University, and the research ethics committee of the four hospitals.

## 3. Results

Study subjects were comprised of 1,038 pregnant women, categorized by BMI into underweight (*n* = 208, 20.0%), normal weight (*n* = 661, 63.7%), and overweight and obese (*n* = 169, 16.3%). The three groups were different in maternal age, maternal height, prepregnancy weight, total weight gain, parity, birth weight, GA at first ANC and ultrasound, and frequency of ANC (*P* < 0.05). Gestational age at delivery, infant's sex, settings, and GA assessment methods were similar (Table 1).

### 3.1. Underweight Pregnant Women

In this group, FH increased from 19.1 cm (±1.7) at 20-week GA to 34.5 cm (±2.3) at 40-week GA. The average increasing rate was 0.8 cm/wk. The highest rate was observed at 1.0 cm/wk between 20 and 32 weeks, declining to 0.6 cm/wk between 33 and 36 weeks, and to 0.2 cm/wk between 37 and 40 weeks (Table 2).

### 3.2. Normal Weight Pregnant Women

The FH in this group increased from 19.1 cm (±1.9) at 20-week GA to 35.4 cm (±2.3) at 40-week GA. The average rate was 0.8 cm/wk, highest between 20 and 32 week at 1.0 cm/wk, declining to 0.8 cm/wk between 33 and 36 weeks, and to 0.2 cm/wk between 37 and 40 weeks (Table 2).

### 3.3. Overweight and Obese Pregnant Women

In this last group, FH increased from 19.2 cm (±2.0) at 20-week GA to 36.2 cm (±2.2) at 40-week GA. The average rate was 0.9 cm/wk, highest between 20 and 32 week at 1.1 cm/wk, declining to 0.7 cm/wk between 33 and 36 weeks, and to 0.2 cm/wk between 37 and 40 weeks (Table 2).

### 3.4. Underweight versus Normal Weight Pregnant Women

At 20 weeks, the FH of the two groups was similar. However, between 33 and 36 weeks, the increasing rate in the underweight was 0.2 cm/wk lower than in the normal weight group, resulting in a difference of 0.9 cm at week 40. Week by week comparisons showed significant differences between weeks 34 and 40 (Table 2).

### 3.5. Overweight and Obese versus Normal Weight Pregnant Women

At 20 weeks, the two groups were also similar in FH. The increasing rate in overweight and obese was 0.1 cm/wk higher until week 32, resulting in a 0.8 cm difference at week 40. Through comparisons by weeks, the FH was significantly different between weeks 30 and 40 (Table 2).

### 3.6. Fundal Height Growth Curve

The FH obtained from quadratic equations allowing for random (individual) effect was estimated by the following equations.

Underweight:

(1) FH (cm)=−19.04386+2.40662 GA (wk) −0.026439 GA2 (wk).

Normal weight:

(2) FH (cm)=−19.61757+2.426414 GA (wk) −0.0260198 GA2 (wk).

Overweight and obese:

(3) FH (cm)=−21.77403+2.552643 GA (wk) −0.0272487  GA2 (wk).

The above equations explained 84%, 86%, and 87% of the variation (*R*-squared = 0.84, 0.86, and 0.87, resp.).

**Figure 1:**
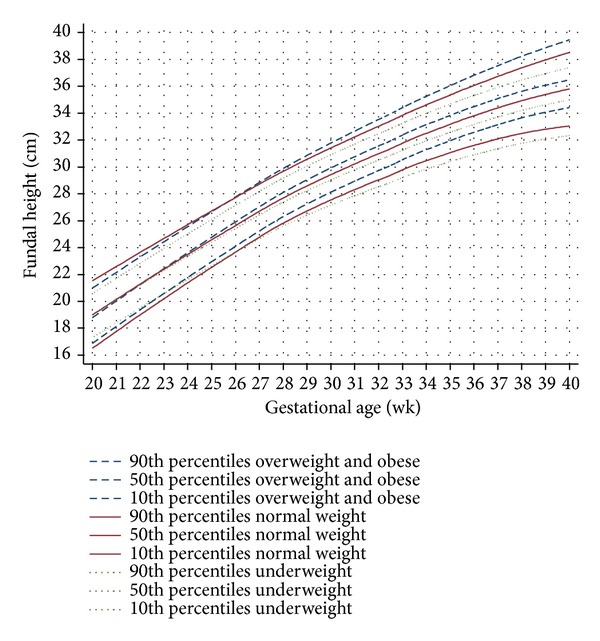
Fundal height growth curve at the 90th, 50th, and 10th percentiles derived from 169 overweight and obese pregnant women (1,281 visits) (dash lines), 661 normal weight pregnant women (4,756 visits) (solid lines), and 208 underweight pregnant women (1,486 visits) (dot lines).

The final FH growth curve of underweight, normal weight, and overweight and obese pregnant women (Figure 1) was presented as smoothed functions of the 10th, 50th, and 90th percentiles derived from Table 3.

Overall comparisons of the FH growth curves among the underweight, the normal weight, and the overweight and obese pregnant women showed that the 50th percentiles of the three groups departed at weeks 22-23. The departures were more obvious at weeks 30–32. The underweight line was 0.1–0.4 cm below the normal line at weeks 23–31 and 0.5–0.8 cm at weeks 32–40. In the opposite direction, the overweight and obese line was 0.1–0.4 cm above the normal line at weeks 22–29 and 0.6–0.8 cm at weeks 30–40 (Figure 1).

The 90th percentile line of the underweight was below the normal weight throughout pregnancy, approximately by 0.4–1.2 cm. The 10th percentile line of the overweight and obese was above the normal weight throughout pregnancy, with the average of 0.4–1.4 cm (Figure 1).

## 4. Discussion

The FH growth curves for the underweight, normal weight, and overweight and obese pregnant women were different regarding the 10th, 50th, and 90th percentiles and the inclining rates per week (Figure 1).

### 4.1. Abdominal Subcutaneous Fat Thickness

Abdominal subcutaneous fat thickness or subcutaneous adipose tissue thickness is directly correlated with FH as FH was measured with nonelastic tapes. Women with abdominal subcutaneous fat thickness were likely to have higher FH than those with thinner abdominal subcutaneous fat. Subcutaneous adipose tissue thickness of anterior abdomen in nonpregnant women with BMI <25, 25–29.9, 30–39.9, and ≥40 kg/m^2^ increased from 10.6 to 17.6, 22.4, and 26.8 mm [12]. Similar correlation was also reported in pregnant women [13]. However, age and number of pregnancies were not directly correlated with abdominal subcutaneous fat thickness [14].

### 4.2. Fetal Weight and Birth Weight

Fetal weight and birth weight (BW) were directly correlated with FH [15, 16] as the size of the uterus expanded to compensate the size of fetus, placenta, and amniotic fluid [17]. Our study excluded pregnancies with any of the above abnormal conditions, as there had been reports that they interfered with FH measurements. As the BW among the three study groups was different (Table 1), this should explain the difference in FH measurements. Beyond these explanations, other determinants of fetal weight and BW were as follows.

#### 4.2.1. Prepregnancy BMI

Prepregnancy BMI influenced fetal weight and BW [18, 19]. Body mass index at the beginning of pregnancy may be considered as a surrogate for the nutritional status of the mothers [20]. In pregnant women with high BMI, altered metabolic hormones, increased placental nutrient transport capacity, and increased nutrient delivery to fetus may result in relatively large fetus. The opposite findings were observed in pregnant women with low BMI [21]. Maternal BMI was also reported to influence fetal growth during the third trimester [20, 22] as a consequence of lowering serum concentrations of insulin-like growth factor binding protein-1 (IGFBP-1) resulting in increased fetal growth [21]. Our study also noticed the more inclining FH among the overweight and obese women (Figure 1).

#### 4.2.2. Gestational Weight Gain

Gestational weight gain was correlated with BW [19, 23]. Large for gestational age fetus or high birth weight infant was common in women with high gestational weight gain. The opposite, small for gestational age fetus or low birth weight infant was also common in women with low gestational weight gain [19, 24], as assessed by the Institute of Medicine (IOM) criteria [11]. The overweight and obese women in our study gained 50.9% more weight than that recommended by the IOM and the underweight women also gained 38.0% less than recommended (Table 1).

#### 4.2.3. Parity

Parity or birth order was positively correlated with BW [25, 26]. Later orders of pregnancy carry residual weight gain and adipose tissue deposit from previous pregnancies. Many studies also reported correlations between parity and both BMI and obesity [27, 28], such that high BW was more prevalent in multiparity and obese women.

#### 4.2.4. Maternal Age

The effect of maternal age on BW varied between studies. Some studies claimed no correlation [29] while some studies reported that age in combination with parity influenced BW, for example, higher parity at younger maternal ages, particularly 15–19 year olds having their second or third birth, appeared to have adverse effects on birth weight [26]. Age may therefore be an effect modifier for BW. In our study, maternal ages of the three weight groups were also different (Table 1). Most of the overweight and obese group was multiparous (72.2%) with the average age of 28.9 ± 5.7 yrs, while most of the underweight group was nulliparous (68.7%) with the average age of 22.4 ± 4.9 yrs.

The above dissimilarities indicated the necessity to develop FH growth curves specifically for women with different body structures. Women with “average” body structure may use a FH growth curve that developed for normal population, while women with slim or obese body shapes should have their own FH growth curves for monitoring and screening abnormal intrauterine growth.

**Figure 2:**
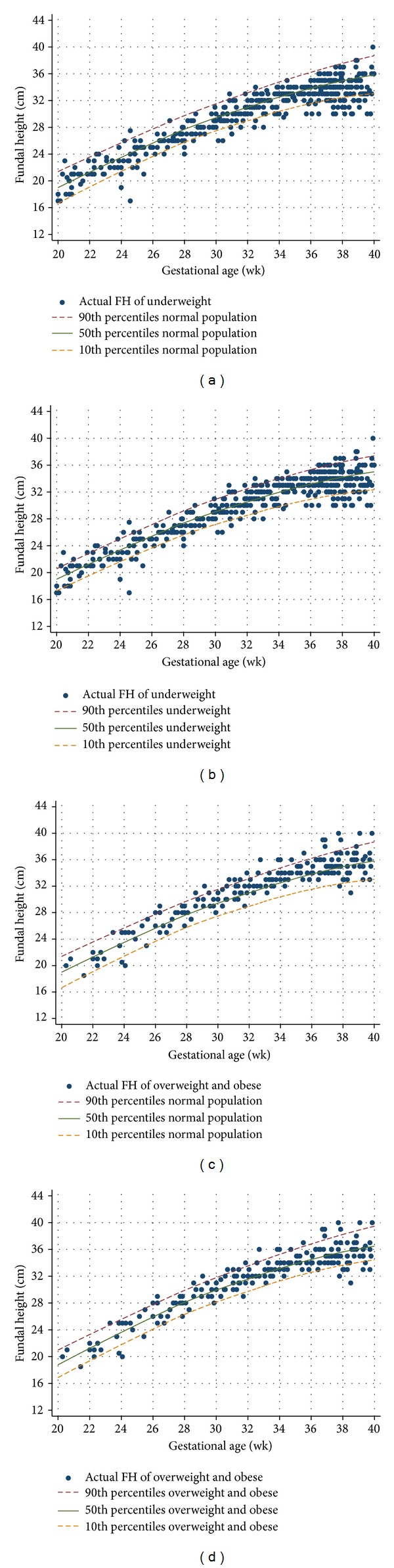
Fundal height (FH) of underweight and overweight and obese pregnant women as screened by different growth curves; (a) underweight pregnant women versus normal population curve; (b) underweight pregnant women versus underweight curve; (c) overweight and obese pregnant women versus normal curve; (d) overweight and obese pregnant women versus overweight and obese curve.

Application of FH growth curves specific for women body shape may reduce an over- or underinvestigation and/or -intervention. For example, in the underweight pregnant women, if a general FH growth curve was applied, FH below the 10th percentiles (size < date) would be detected in 15.4%, and FH above the 90th percentiles (size > date) in 1.2% (Figure 2(a)). On the contrary, if a specific FH growth curve for this group were applied the FH below the 10th percentiles would have been detected in 11.4%, and that above the 90th percentiles would have been 5.8% (Figure 2(b)). As a consequence, size < date was reduced 4.0% and size > date was increased 4.6%.

In the overweight and obese pregnant women, if a general FH growth curve was applied, FH above the 90th percentiles (size > date) would be detected in 11.1% and that below the 10th percentiles (size < date) in 3.0% (Figure 2(c)). If a specific FH growth curve for this group was applied the FH above the 90th percentiles would have been detected in 9.0%, and that below the 10th percentiles would have been 9.0% (Figure 2(d)), resulting in a 2.1% reduction of size > date and a 6.0% increase of size < date.

Body mass index categorization in the present study followed the WHO criteria [9], but the obese group was combined with the overweight group. This may limit the use of the developed curve in women with very high BMI (obese class II, BMI 35.0–39.9 kg/m^2^ or obese class III, BMI ≥ 40.0 kg/m^2^). Validation of the developed curve should be done before applying into routine clinical practice.

However, the developed FH growth curves for the underweight and overweight and obese pregnant women in this study was based on routine ANC practice of the four university affiliated hospitals in the upper northern part of Thailand. Generalization to other settings with different context, including the methods of FH measurement and the methods of GA assessments, may be limited.

Furthermore, the measurement of FH in normal practice is still considered “subjective” to intraobserver and interobserver errors. A standardized method should be reinforced, such as frequent validation or calibration, as we believe that simple FH measurement is of great value as a screening tool for routine antenatal care practice, especially in areas where health resources are limited.

## 5. Conclusions

Fundal height growth curves of the underweight (BMI < 18.5 kg/m^2^) and overweight and obese (BMI ≥ 25.0 kg/m^2^) women were different from the normal weight. In monitoring or screening for abnormal intrauterine growth in slim or obese women, FH growth curves specifically developed for such women should be applied. This may reduce an over- or underinvestigation and/or -intervention as a consequence of an inappropriate application of FH growth curve for normal weight women.

## Figures and Tables

**Table 1:** Characteristics of pregnant women.

Characteristics	Prepregnancy BMI (kg/m^2^)						Global *P* value*
	Underweight (<18.5)		Normal weight (18.5–24.9)		Overweight and obese (≥25.0)		
	(*n* = 208; 1,486 visits)		(*n* = 661; 4,756 visits)		(*n* = 169; 1,281 visits)		
	Mean	±SD	Mean	±SD	Mean	±SD	
Maternal age (year)	22.8	±5.0	25.9	±6.3	28.1	±5.7	<0.001
Maternal height (cm)	157.5	±6.1	155.6	±5.4	156.1	±5.6	0.011
Prepregnancy weight (kg)	42.9	±4.0	51.5	±5.4	69.0	±8.0	<0.001
Prepregnancy BMI (kg/m^2^)	17.3	±1.1	21.2	±1.8	28.3	±2.7	<0.001
Total weight gain (kg)	13.9	±4.0	13.9	±4.5	11.2	±5.2	<0.001
Gestational weight gain** (*n*, %)							<0.001
Less than recommended	79	38.0	197	29.9	27	16.0	
Within recommended	99	47.6	273	41.4	56	33.1	
More than recommended	30	14.4	189	28.7	86	50.9	
Parity (*n*, %)							<0.001
Nulliparous	143	68.7	333	50.4	47	27.8	
Multiparous	65	31.3	328	49.6	122	72.2	
GA at delivery (wk)	39.1	±1.1	39.2	±1.1	39.3	±1.1	0.107
Infant's sex (*n*, %)							0.918
Female	101	48.6	294	44.5	84	49.7	
Male	107	51.4	367	55.5	85	50.3	
Birth weight (gm)	3,035.0	±318.4	3,126.5	±327.0	3,201.0	±302.3	<0.001

Settings (*n*, %)							0.754
Secondary care hospitals	140	67.3	445	67.3	111	65.7	
Tertiary care hospitals	68	32.7	216	32.7	58	34.3	
Gestational age by (*n*, %)							0.051
LMP	115	55.3	424	64.2	109	64.5	
Ultrasound	93	44.7	237	35.8	60	35.5	
GA at first ANC (wk)	14.2	±5.2	12.7	±4.8	12.6	±4.9	0.001
GA at ultrasound (wk)	16.9	±5.4	15.8	±5.1	15.2	±5.3	0.037
Frequency of ANC (per woman)	6.9	±2.2	7.3	±2.1	7.5	±2.2	0.020

BMI: body mass index; GA: gestational age; LMP: last menstrual period; ANC: antenatal care.

*P value from nonparametric tests for trend.

**Recommendations by the Institute of Medicine (2009) [11]: underweight prepregnancy BMI (<18.5 kg/m^2^) = 12.5–18 kg; normal weight (18.5–24.9 kg/m^2^) = 11.5–16 kg; overweight (25.0–29.9 kg/m^2^) = 7–11.5 kg; obese (≥30.0 kg/m^2^) = 5–9 kg.

**Table 2:** Mean and standard deviation of fundal height (in centimeters) for each gestational age in underweight, normal weight, and overweight and obese pregnant women.

GA (wk)	Fundal height (cm)							
	Underweight (*n* = 208; 1,486 visits)		Normal weight (*n* = 661; 4,756 visits)		*P* value*	* *Overweight and obese (*n* = 169; 1,281 visits)		*P* value**
	Number	Mean ± SD	Number	Mean ± SD		Number	Mean ± SD	
20	35	19.1 ± 1.7	104	19.1 ± 1.9	0.933	27	19.2 ± 2.0	0.867
21	31	20.3 ± 1.8	90	20.4 ± 1.9	0.780	23	20.2 ± 1.8	0.628
22	23	21.4 ± 2.5	72	21.3 ± 2.1	0.874	24	21.9 ± 1.6	0.276
23	25	22.7 ± 1.6	83	22.4 ± 1.9	0.444	15	22.4 ± 1.9	0.901
24	53	23.2 ± 1.7	167	23.8 ± 1.6	0.020	46	23.8 ± 2.1	0.959
25	51	24.4 ± 1.9	145	24.4 ± 1.8	0.808	39	24.5 ± 1.7	0.896
26	22	24.9 ± 1.5	73	25.2 ± 1.5	0.350	31	25.9 ± 2.0	0.054
27	32	26.3 ± 1.5	90	26.7 ± 1.7	0.259	20	26.8 ± 2.0	0.957
28	62	27.1 ± 1.8	225	27.7 ± 1.7	0.034	62	28.1 ± 1.8	0.077
29	54	28.3 ± 1.9	167	28.7 ± 1.8	0.138	47	28.8 ± 1.8	0.692
30	66	29.1 ± 1.7	223	29.7 ± 1.6	0.018	63	30.5 ± 1.4	0.001
31	73	30.1 ± 1.7	209	30.4 ± 1.7	0.240	54	30.9 ± 1.5	0.050
32	87	31.0 ± 1.7	278	31.4 ± 1.6	0.045	73	32.2 ± 1.5	<0.001
33	70	31.8 ± 1.7	249	32.1 ± 1.7	0.231	67	32.9 ± 1.4	<0.001
34	89	32.4 ± 1.8	249	33.2 ± 1.5	<0.001	71	33.6 ± 1.6	0.048
35	73	33.3 ± 1.5	251	33.7 ± 1.6	0.082	67	34.6 ± 1.7	<0.001
36	94	33.7 ± 1.8	297	34.5 ± 1.5	<0.001	82	34.9 ± 1.8	0.061
37	145	34.0 ± 2.0	481	34.9 ± 1.8	<0.001	113	35.7 ± 1.7	<0.001
38	164	34.4 ± 2.0	529	35.0 ± 1.8	0.003	147	36.0 ± 2.1	<0.001
39	151	34.3 ± 2.0	495	35.2 ± 2.3	<0.001	116	36.1 ± 2.3	<0.001
40	86	34.5 ± 2.3	279	35.4 ± 2.3	0.002	94	36.2 ± 2.2	0.002

GA: gestational age.

*Underweight versus normal weight.

**Overweight and obese versus normal weight.

**Table 3:** Coefficients at the 10th, 50th, and 90th percentiles for fundal height prediction equations in underweight, normal weight, and overweight and obese pregnant women from multilevel models.

Parameters	Coefficient (cm)		
	10th percentiles	50th percentiles	90th percentiles
Underweight			
Constant	−22.31506	−18.08827	−16.36419
GA (wk)	2.550561	2.345531	2.314052
GA^2^ (wk)	−0.029636	−0.025478	−0.024279
Normal weight			
Constant	−28.94898	−19.02109	−10.01752
GA (wk)	2.943811	2.392514	1.914523
GA^2^ (wk)	−0.034877	−0.025569	−0.017541
Overweight and obese			
Constant	−26.51847	−22.88809	−13.76647
GA (wk)	2.769345	2.639944	2.115199
GA^2^ (wk)	−0.031173	−0.028917	−0.019630

GA: gestational age.
